# In Vitro Evaluation of Curcumin- and Quercetin-Loaded Nanoemulsions for Intranasal Administration: Effect of Surface Charge and Viscosity

**DOI:** 10.3390/pharmaceutics14010194

**Published:** 2022-01-14

**Authors:** Gustavo Vaz, Adryana Clementino, Evgenia Mitsou, Elena Ferrari, Francesca Buttini, Cristina Sissa, Aristotelis Xenakis, Fabio Sonvico, Cristiana Lima Dora

**Affiliations:** 1 Laboratório de Nanotecnologia Aplicada à Saúde, Programa de Pós-Graduação em Ciências da Saúde, Universidade Federal do Rio Grande, Rio Grande 96210-900, RS, Brazil; richtervaz@gmail.com (G.V.); cristianadora@gmail.com (C.L.D.); 2 Food and Drug Department, University of Parma, 43124 Parma, PR, Italy; adryanarc@gmail.com (A.C.); francesca.buttini@unipr.it (F.B.); 3 Institute of Chemical Biology, National Hellenic Research Foundation, 11635 Athens, Greece; emitsou@eie.gr (E.M.); arisx@eie.gr (A.X.); 4 Department of Chemistry, Life Sciences, and Environmental Sustainability, University of Parma, 43124 Parma, PR, Italy; elena.ferrari2@unipr.it (E.F.); cristina.sissa@unipr.it (C.S.)

**Keywords:** curcumin, quercetin, electron paramagnetic resonance spectroscopy, central nervous system, nose-to-brain delivery, RPMI 2650

## Abstract

The nose-to-brain delivery of neuroprotective natural compounds is an appealing approach for the treatment of neurodegenerative diseases. Nanoemulsions containing curcumin (CUR) and quercetin (QU) were prepared by high-pressure homogenization and characterized physicochemically and structurally. A negative (CQ_NE−), a positive (CQ_NE+), and a gel (CQ_NEgel) formulation were developed. The mean particle size of the CQ_NE− and CQ_NE+ was below 120 nm, while this increased to 240 nm for the CQ_NEgel. The formulations showed high encapsulation efficiency and protected the CUR/QU from biological/chemical degradation. Electron paramagnetic resonance spectroscopy showed that the CUR/QU were located at the interface of the oil phase in the proximity of the surfactant layer. The cytotoxicity studies showed that the formulations containing CUR/QU protected human nasal cells from the toxicity evidenced for blank NEs. No permeation across an in vitro model nasal epithelium was evidenced for CUR/QU, probably due to their poor water-solubility and instability in physiological buffers. However, the nasal cells’ drug uptake showed that the total amount of CUR/QU in the cells was related to the NE characteristics (CQ_NE− > CQ_NE+ > CQ_NEgel). The method used allowed the obtainment of nanocarriers of an appropriate size for nasal administration. The treatment of the cells showed the protection of cellular viability, holding promise as an anti-inflammatory treatment able to prevent neurodegenerative diseases.

## 1. Introduction

The nasal administration is a promising non-invasive route for the delivery of active compounds. The human nasal cavity has a surface area of approximately 150 cm^2^, with a total volume of 15–20 mL and an extensive vascularization with a leaky epithelium, providing an interesting region for the absorption of delivery drugs [1,2]. Drugs administrated in the nasal cavity can perform a local activity, a systemic action, or can be targeted to the brain [3,4].

The nasal administration can provide a rapid absorption of compounds as well as an increase in bioavailability for therapeutic molecules that have poor oral absorption or a high pre-systemic metabolism, due to the avoidance of the first-pass effect [5,6,7]. Moreover, due to the presence of the olfactory and trigeminal nerves, the nasal cavity is in direct contact with the central nervous system (CNS) [8,9].

Despite these advantages, the use of this route of administration presents some limitations, such as the short residence time of the compounds and formulations in the nasal cavity to mucociliary clearance (10–20 min), the presence of enzymatic activity, and the restricted volume/dose than can be administered (25–200 µL) [1,10]. Aiming to overcome these limitations, the development of NEs with the ability to enhance the residence time in the nasal cavity, protect the compounds from a possible enzymatic degradation, and improve the permeation through the nasal neuroepithelium appears as a viable approach for the brain delivery of pharmacologically active compounds [11,12].

Natural products, such as curcumin (CUR) and quercetin (QU), have been indicated as promising compounds for the treatment of neurodegeneration because of their peculiar pharmacological and biological activities [13]. Curcumin (CUR) presents neuroprotective potential by reason of its anti-amyloid, anti-oxidant, and anti-inflammatory properties [14,15], while QU was demonstrated to protect neurons from oxidative damage (reducing the lipid peroxidation), to prevent amyloid-β fibrils formation, to reduce cell lysis, and to counterbalance inflammatory cascade pathways [16]. However, the potential clinical use of these promising natural compounds is hampered by their poor aqueous solubility, extensive metabolization, and limited bioavailability.

Nanotechnology-based delivery systems can enable the use of natural compounds in the treatment of neurodegenerative diseases [17]. Han and collaborators showed that nanocarriers with QU had therapeutic potential for the treatment of neurodegenerative diseases due to the inhibition of β-amyloid aggregation and scavenging of free radicals [18]. Tiwari and collaborators concluded in their study that CUR-loaded nanoparticles induced the proliferation of neural stem cells and neuronal differentiation in vitro and in the hippocampus and subventricular zone of adult Wistar rats after intraperitoneal injection [19].

The objectives of the present work were the development, physicochemical and structural characterization, and evaluation of the toxicity of three different NEs loaded with CUR and QU on a model of nasal mucosa. Finally, the permeation-enhancing properties of these NEs when compared to the free forms of the two natural compounds through the same model of the nasal epithelium was assessed. A “classic” negatively charged nanoemulsion (CQ_NE−) was modified in order to modulate its physicochemical properties to investigate any improvement in the nasal permeation. The addition of a cationic agent was used to obtain the positive formulation (CQ_NE+) to enhance the mucoadhesion and possibly to enhance penetration/permeation through the nasal mucosa. At the same time, a gel formulation (CQ_NEgel) was prepared with the addition of an in situ gelling agent to prolong the residence time in the nasal cavity. Special focus was put on a structural investigation to shed more light on the interactions of the formulation components that affect the performance of a designed delivery system and on the ability to follow the kinetics of the pro-inflammatory oxidant species scavenging reaction.

## 2. Materials and Methods

### 2.1. Materials

Curcumin, quercetin, PEG 660-stearate, castor oil, dimethyl sulfoxide, cetalkonium chloride, and deacetylated gellan gum (Gelzan^®^ CM) were purchased from Sigma-Aldrich (St. Louis, MO, USA). Egg lecithin containing 80% phosphatidylcholine (Lipoid E80^®^), and purified fish oil (DHA/EPA) were purchased from Lipoid (Ludwigshafen, Germany). The methanol and acetonitrile of HPLC grade used in the analysis were purchased from Panreac^®^ (Barcelona, Spain). Benzalkonium chloride was provided by Acef Spa (Fiorenzuola d’Arda, Italy). The spin probes, 5-doxyl stearic acid (5-DSA) and 12-doxyl stearic acid (12-DSA), were produced by Sigma-Aldrich (St. Louis, MO, USA). The free radical Tempol ((2,2,6,6-tetramethylpiperidin-1-yl)oxyl) was purchased from Alfa Aesar (Ward Hill, MA, USA). Centrifugal filter devices (Vivaspin^®^ 2; 30,000 Da molecular weight cut-off, Hydrosart^®^ cellulose) were obtained from Sartorius (Göttingen, Germany). Cell line RPMI 2650 (CCL-30) was purchased from American Type Culture Collection (ATCC) (Manassas, VA, USA). Minimum essential medium (MEM), fetal bovine serum (FBS), and collagen I bovine protein were acquired from Gibco™ (Thermo Fisher Scientific, Waltham, MA, USA). Trypsin–EDTA and Penicillin–Streptomycin solutions were purchased from Aurogene (Rome, Italy). The 75 cm^2^ flasks, polystyrene and Transwell^®^ cell culture inserts (1.13 cm^2^ polyester membrane, 0.4 µm pore size, 12 mm insert), and 12-well polystyrene plates were obtained from Corning Costar (Corning, NY, USA). The 48-well black plates (SensoPlate™ Black) were obtained from Greiner Bio-One International (Kremsmünster, Austria). All other chemicals were of analytical grade. Ultrapure and degassed ultrapure water (Purelab Flex; ELGA LabWater, High Wycombe, UK) was used in all experiments.

### 2.2. Production of Nanoemulsions

NEs were formed via high-energy emulsification followed by high-pressure homogenization of a mixture of an aqueous phase and an oil phase, as previously published by our group [20]. To prepare the NE loaded with CUR and QU, the oil and aqueous phases of the emulsion were firstly prepared separately. To prepare the water phase, the surfactant PEG 660-stearate was dissolved in ultrapure water (1.5% *w*/*v*). The oil phase containing castor oil, purified fish oil (DHA/EPA), and egg lecithin (Lipoid E80^®^) was maintained for 30 min at 68 °C under magnetic stirring at 1500 rpm. CUR and QU were dissolved at an optimal concentration determined in a previous work [15]. Then, the aqueous phase (60 mL), previously heated to 80 °C, was then added to the oil phase under magnetic stirring at 1500 rpm, following 2 min of mixing. After adding the aqueous phase to the oil phase, the dispersion was further homogenized for 2 min using a mechanical high-performance dispersing device (Ultraturrax TP 18/10–10N; IKA-Werke GmbH, Staufen, Germany) at 14,500 rpm to form the pre-emulsion. Finally, the pre-emulsion was passed through a high-pressure homogenizer (PandaPLUS 2000 Laboratory Homogenizer, GEA Niro Soavi, Parma, Italy) for 13 cycles of 20 s each at 1000 bars, totaling 4 min and 20 s. For the preparation of CQ_NE, the CUR and QU compounds were added to the organic phase of the formulation and maintained under heating (68 °C) and stirring (1500 rpm) for 30 min (Table 1), prior to formulating.

To convert the negative charge of the NEs to positive, a formulation optimization was carried out by varying the type and the amount of a cationic agent to define the minimum amount of cationic agent to be added to the formulation to obtain a positively charged emulsion (data not shown). Finally, cetalkonium chloride (CC) was selected. In brief, for the preparation of the positive formulations, the cationic surfactant, i.e., CC (0.0175% *w*/*v*), was added to the aqueous phase of the formulation due to its hydrophilic character and heated until 80 °C. To prepare the NEs containing the gelling agent Gelzan, a deacetylated form of gellan gum was added (0.5%, *w*/*v*) to the formulation after the preparation process described above and kept under magnetic stirring at 700 rpm at 68 °C for 5 min.

### 2.3. Physicochemical Characterization of CUR/QU-Loaded NEs

#### 2.3.1. Nanoemulsions Particle Size and Surface Charge

The particle size and polydispersity index (PDI) of the NEs were measured using dynamic light scattering (DLS) (Zetasizer Nano ZS, Malvern Panalytical, Malvern, UK). In order to prepare samples for DLS measurements, the nanoemulsions were diluted 1:100 with ultrapure water. The measurements were carried out in triplicate runs at a scattering angle of 173° and temperature of 25 °C. The surface charge of the nanoemulsions was measured as zeta potential using Phase Analysis Light Scattering (PALS) with the same instrument. For PALS measurements, nanoemulsions were diluted 1:100 with ultrapure water to achieve a mean count rate of around 400 kcps and a conductance of around 300 μS. Zeta potential values were mean of three runs per sample.

#### 2.3.2. Nanoparticle Tracking Analysis

In order to further characterize the particle size distribution and to determine droplets’ concentration in suspension, nanoparticle tracking analysis (NTA) experiments were conducted using a NanoSight NS300 (Malvern Pananalytical, Malvern, UK) instrument equipped with a 480 nm laser light source. A 20× magnification microscope was used for the particle tracking with a field of view of approximately 100 × 80 × 10 μm. The built-in sCMOS camera was used to record videos, and the particle tracking was analyzed by NTA 3.1 software (Malvern Pananalytical, Malvern, UK).

NTA tracks single particles in Brownian motion using the light they scatter. Videos of the particles’ tracks, projected on the x–y plane, were analyzed by the built-in NTA software. The center of each individual particle moving in the observation volume was located and tracked, determining the mean square displacement by each particle in the x and y directions. The mean square displacement is then converted into particle size on the basis of a variation of the Stokes–Einstein equation (Equation (1)) considering that the motion is tracked in two dimensions:

(1) $${\left(x,\;y\right)}^{2}=\frac{4\mathrm{TkB}}{3\pi \eta d_{h}}$$

where kB is the Boltzmann constant and (x, y)^2^ is the mean squared displacement of a particle during time t at temperature T, in a medium of viscosity η, with a hydrodynamic diameter of d_h_. Knowing the volume of the suspension and the dilution, the associated NTA software calculates an approximate concentration of the nanoparticles inside the colloidal suspension [21,22].

Only blank NEs were analyzed by NTA since this first NE preparation stands as the platform for all the proposed formulations. Moreover, curcumin- and quercetin-loaded NE fluorescence interfered with the analysis of the laser available in-house for the NTA measurements.

The NE was diluted 1:10,000 with ultrapure water to allow the tracking of individual particles. After that, sample was drawn into a 1 mL plastic syringe, which was used for full-sample injection into the instrument sample chamber. The nanoparticle images were acquired using a video capture mode of the sample for three 60 s analyses, which were used for subsequent analysis. Measurement was carried out at a defined temperature (28.7–28.9 °C) and viscosity (0.815–0.818 cP). The results were obtained as mean and SD of three runs.

#### 2.3.3. Determination of Curcumin and Quercetin Concentration by HPLC

The CUR and QU content and the entrapment efficiency (%) were analyzed using a validated HPLC method published previously [20]. Briefly, the HPLC system consisted of a pump LC-10AS (Shimadzu Cor., Kyoto, Japan), a UV-VIS detector SPD-10A, (Shimadzu Cor., Kyoto, Japan), and an auto sampler model 542 (ESA, Chelmsford, MA, USA). The experiments were conducted using a reversed-phase C18 column (Symmetry 75 × 4.6 mm I.D., with a particle size of 3.5 µm, Waters Corp., Milford, MA, USA), maintained at 35 ± 1 °C. The mobile phase consisted of a 1% phosphoric acid: acetonitrile mixture (40:60 *v*/*v*; pH 2.6) and was eluted under isocratic conditions at a flow rate of 1.2 mL/min for 15 min. The detector was set at 400 nm, and the injection volume was 10 µL.

The HPLC method was validated according to the International Conference for Harmonization of Technical Requirements for Registration of Pharmaceuticals for Human Use ICH-Q2 (2005), using the following parameters: specificity, linearity, accuracy, precision, and determination of the limits of detection (LOD) and quantification (LOQ). The calibration for CUR and QU was linear over the range of 0.5–10.0 µg/mL (R^2^ = 0.9999) for both compounds. For HPLC analysis, an aliquot of 50 µL of the NEs was completely dissolved (dilution 200 times) with methanol: water (50:50 *v*/*v*; pH 2.6). The CUR and QU total concentration in the colloidal suspensions were calculated after determining the drug concentration in the suspension and was expressed in μg of CUR/QU per mL of preparation. The CUR and QU recovery were calculated as the percentage of the total drug concentration found in the nanocarrier in relation to the initially added amount. The entrapment efficiency (%) was estimated indirectly as the difference between the total concentration of CUR/QU of the nanocarrier and the ultrafiltrate concentration. The filtrate was obtained by an ultrafiltration/centrifugation method of an aliquot (500 µL) of the NEs using a centrifugal concentrator (Vivaspin^®^ 2; 30,000 molecular weight cut-off, Hydrosart^®^ cellulose) from Sartorius (Göttingen, Germany). All samples were analyzed in triplicates [20].

#### 2.3.4. Chemical Stability of Curcumin and Quercetin in Nanoemulsions

The stability of curcumin and quercetin content in the positively (CQ_NE+) and negatively charged nanoemulsion (CQ_NE−) stored in refrigerated conditions (2–8 °C) was assessed by determining the two natural compounds’ content in the formulation at predetermined time points by HPLC for up to 30 and 120 days, respectively.

The photostability of curcumin in the same nanoemulsions was determined using curcumin solution in acetonitrile as a control. Briefly, a Hellma^®^ Ultra Micro Cuvette (Suprasil^®^ quartz, aperture 1.5 × 5 mm, volume 12 μL; Merck, Darmstadt, Germany) was filled either with a 4 × 10^−4^ M curcumin solution in acetonitrile or with CQ_NE− and CQ_NE+ formulations diluted 1:1000 in water and was exposed to 410 nm wavelength light in a Fluoromax-3 fluorimeter (Horiba Jobin Yvon, Palaiseau, France). The cuvette was transparent only in the aperture. This configuration ensured complete irradiation of the solution and excluded diffusion effects due to incomplete irradiation, as the light beam was wider than the aperture. The power of the light transmitted through the aperture of the empty cuvette was 0.36 mW. The photodegradation of curcumin was monitored by absorption spectroscopy. The measurements were performed with a PerkinElmer Lambda 650 UV/vis spectrophotometer (PerkinElmer Italia, Milan, Italy), after 3, 8, 15, 25, and 40 min of exposure, in single ray mode.

#### 2.3.5. Electron Paramagnetic Spin Resonance Spectroscopy

In the present study, electron paramagnetic resonance (EPR) spectroscopy, applying the spin-probing technique, was used to investigate the interfacial properties of NEs with different surface charges in the absence (NE− and NE+, Table 1) and the presence (CQ_NE− and CQ_NE+, Table 1) of the poorly water-soluble antioxidants, CUR and QU. In this study, only the negative and positive nanoemulsions were employed for the investigations, since the negative NE− was used as the platform for the preparation of the gelled nanoemulsion.

The doxyl free radical, 5-doxyl stearic acid [5-(1-oxyl-2,2-dimethyl-oxazolidin) stearic acid, 5-DSA] was used as a membrane spin probe (Table 2). Being a fatty acid, 5-DSA has an amphiphilic nature deriving from the presence of a polar head group (-COOH) and a hydrophobic moiety. Due to the structure of 5-DSA and especially of the location of the nitroxide paramagnetic ring (doxyl group) at the 5th carbon atom of the hydrocarbon chain, the unpaired electron is located closer to the polar head of the fatty acid and consequently closer to the surfactant/co-surfactant polar head group area [23,24]. In addition, 16-doxyl stearic acid [2-(14-carboxytetradecyl)-2-ethyl-4,4-dimethyl-3-oxazolidinyloxy, 16-DSA] was used in the present study in order to investigate two different depths of the NE’s surfactant layer. In that case, the doxyl group is located at the 16th carbon atom of the hydrocarbon chain and the unpaired electron is located in the part of the surfactant layer closer to the oil phase of the system. Those interface-located fatty acid spin probes give EPR spectra reflecting the rigidity/flexibility of their environment from the depth of the membrane where the doxyl ring is located. As a result, with the use of the above spin probes (i) alterations caused in the membrane in the positive nanoemulsion and (ii) the localization of the encapsulated compounds in the NE systems were both evaluated.

##### Preparation of the Samples

To obtain the desired concentration of the spin probes in the NEs, 1 mL of each NE was added to a tube into which the appropriate amount of the spin probe had been deposited previously. More specifically, 15 μL of an ethanolic stock solution of 5-DSA or 16-DSA (7.8 × 10^−3^ M) were transferred in the tube and the solvent was allowed to evaporate at room temperature. The samples remained overnight in a water bath (25 °C) in order to reach equilibrium. Final concentration of 5-DSA or 16-DSA in the NEs was 0.117 mM.

##### EPR Measurements

Electron paramagnetic resonance (EPR) spectra were recorded at constant room temperature (25 °C) using a Bruker EMX EPR spectrometer (Billerica, MA, USA). Experiments were conducted with the use of a WG-813-Q Wilmad (Buena, NJ, USA) Suprasil flat cell. Typical instrument settings were: center field 0.349 T, scan range 0.01 T, gain 2.24 × 103, time constant 5.12 ms, modulation amplitude 0.4 mT, and frequency 9.78 GHz. Data collection and analysis were performed using the BrukerWinEPR acquisition and processing program.

##### Electron Paramagnetic Spin Resonance Studies

In the present study, EPR spectra of 5-DSA and 16-DSA were analyzed to provide information regarding molecular motion, order, and polarity across the surfactants’ monolayer. For this purpose, rotational correlation time (τ_R_), order parameter (S), and isotropic hyperfine splitting constant (α′_0_) were calculated from the obtained spectra as described below.

To express the mobility of each probe, we use the τ_R_ parameter, which is expressed in nanoseconds and reflects the mobility of the probe in the membrane.

##### Calculation of the Rotational Correlation Time (τ_R_)

**Figure 1:**
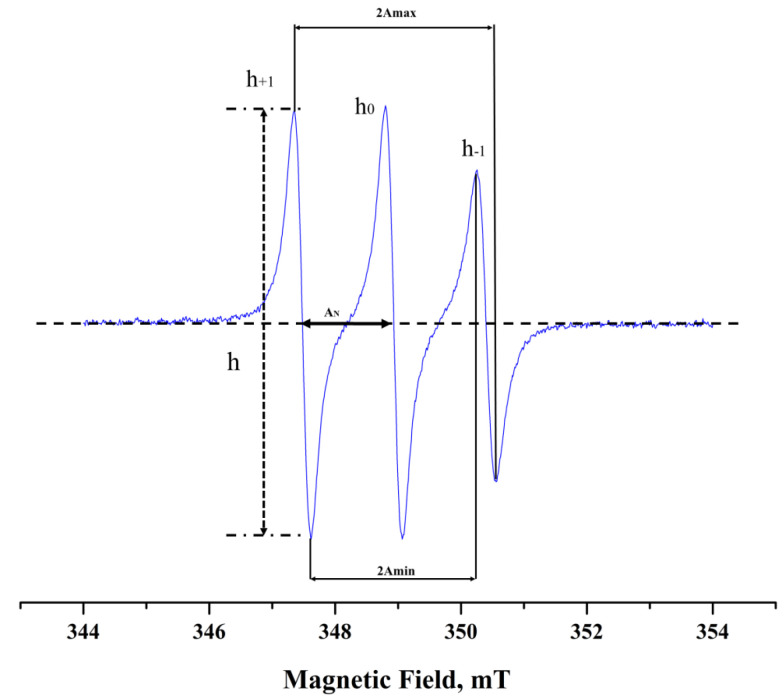
EPR spectrum of 5-DSA in isopropanol.

The rotational correlation time, τ_R_, of the spin probe was calculated from the EPR spectra using the following relationship (Equation (2)):

(2) $$\tau_{R}=\left(6\times 10^{-10}\right)\left[{\left(\frac{h_{0}}{h_{+1}}\right)}^{\frac{1}{2}}+{\left(\frac{h_{0}}{h_{-1}}\right)}^{\frac{1}{2}}-2\right]\Delta H_{0}\left(s\right)$$

where ΔH_0_ is the width of the central peak and h_+1_, h_0_, and h_−1_ are the heights of the peaks from the low to the high field, respectively, in the characteristic 3-lined spectrum of nitroxides (Figure 1). The above-mentioned relationship is applicable only in the fast motion region (τ_R_ < 3 ns) where the spin-probe molecules undergo an isotropic motion in an isotropic environment. For τ_R_ values in the slow-motion region (τ_R_ > 3 ns) the calculations are based on computer simulations assuming average τ_R_. In the present study, the equations were used for the 16-DSA as the motion of the spin probe belongs to the fast-motion regime. In the case of 5-DSA, spectral simulations were performed with home-written programs in MATLAB (MathWorks, Natick, MA, USA) employing the EasySpin toolbox for EPR spectroscopy.

##### Calculation of the Order Parameter (S) and the Isotropic Hyperfine Splitting Constant (α′_0_)

From the spectral characteristics, we have calculated two more parameters: the order parameter, S, and the isotropic hyperfine splitting constant, α′_0_ [23,25].

S is defined as (Equation (3)):

(3) $$S=\left(A_{\mathrm{II}}-A_{⊥}\right)/\left[A_{\mathrm{ZZ}}-\left(\frac{1}{2}\right)\left(A_{\mathrm{XX}}+A_{\mathrm{YY}}\right)\right]\left(\alpha_{0}/\alpha_{N}\right)$$

where A_II_ corresponds to the half-distance of the outer maximum hyperfine splitting, 2A_max_, and A_⊥_ is calculated from the following equation (Equation (4)):

(4) $$A_{⊥}=A_{\min}=1.4\left(1-S^{\mathrm{app}}\right)\;\mathrm{and}\;S^{\mathrm{app}}=\left(A_{\max}-A_{\min}\right)/\left[A_{\mathrm{ZZ}}-\left(\frac{1}{2}\right)\left(A_{\mathrm{XX}}+A_{\mathrm{YY}}\right)\right]$$

where A_min_ is equal to the half-distance of the inner minimum hyperfine splitting. A_XX_, A_YY_, and A_ZZ_ are the single-crystal values of the spin probe equal to 0.63, 0.58, and 3.36 mT, respectively, and are indicative for doxyl derivatives.

The ratio (α_0_/α′_0_) is the polarity correction where α_0_ = (A_YY_ + A_XX_ + A_ZZ_)/3 and α′_0_ is the isotropic hyperfine splitting constant for the spin probe in the membrane and is defined as (Equation (5)):

(5) $$\alpha_{0}^{\prime }=\frac{A_{\mathrm{II}}+2A_{⊥}}{3}\;$$

Order parameter S provides a measure of the spin probe’s arrangement in a supramolecular assembly and varies from 0 to 1, with S = 1 for the completely ordered state and S = 0 for the completely random state.

The hyperfine splitting constant, reflecting the polarity in the environment of the doxyl paramagnetic ring, can be also calculated directly from the EPR spectrum. α′_0_ value is taken as the distance between the first and the second line (peak) of the first derivate of the EPR spectrum (Figure 1). The α′_0_ value is sensitive to the polarity of the environment of the spin probe and is increased when the polarity of the medium is increased.

##### Kinetics of the Scavenging Reaction

**Figure 2:**
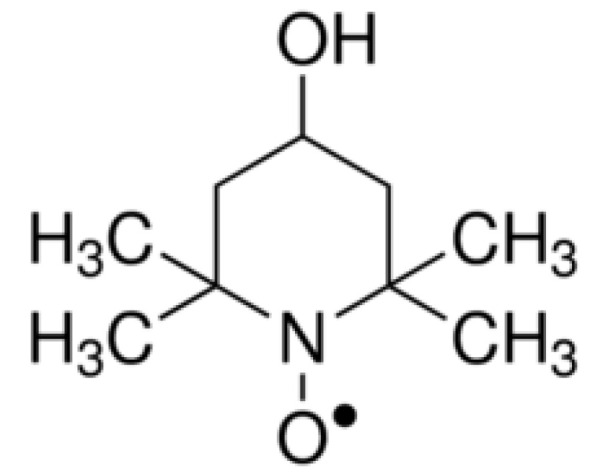
Chemical structure of 4-Hydroxy-Tempo (Tempol).

The EPR antioxidant activity tests permit the detection of the direct effect of an antioxidant on a free radical. In addition, there are no medium restrictions as both hydrophilic and lipophilic radicals can be used. Consequently, the kinetics of the scavenging reaction can be evaluated. For the antioxidant assessment, in order to observe the scavenging effect not only of encapsulated antioxidants such as CUR and QU but also of the developed systems, stable free radicals are utilized in combination with EPR. In the present study, we assessed the antioxidant activity of empty and loaded high-energy NEs using the EPR technique and the hydrophilic free radical Tempol (see Figure 2). A typical reaction mixture contained 90% *v*/*v* 1 mM Tempol aqueous solution and 10% *v*/*v* sample. The mixture was stirred and immediately transferred into an EPR cell for analysis. EPR spectra were taken at room temperature for 30 min at given time intervals. When the stable free radical Tempol was dissolved in the reaction mixture, a well-defined EPR spectrum consisting of three peaks was obtained. The % inhibition of the EPR spectrum was calculated from the following equation (Equation (6)):

(6) $$\%\;\mathrm{Inhibition}=\left(1-\frac{A}{A_{0}}\right)\cdot 100$$

where A_0_ is the integral intensity of the EPR spectrum of a control sample and A (1 mM of the free radical) is the integral intensity of the EPR spectrum in the presence of the sample.

EPR spectrum of Tempol is reduced after the scavenging reaction between this stable free radical and the antioxidant (Equation (7)):

(7) $$\mathrm{Tempol}+H-A\to \mathrm{Tempol}-A+A^{●}$$

where H − A is a hydrogen-donating antioxidant compound, Tempol-H is the resulting EPR silent hydroxylamine, and A^●^ is the resulting unstable radical [26].

All experiments were performed in triplicate.

### 2.4. Cytotoxicity Assay

For cell culture experiments, RPMI 2650 cells, an immortalized human nasal cell line derived from an anaplastic squamous cell carcinoma of the human nasal septum, were used between passages 31 and 42. RPMI 2650 cells have been proposed as a suitable model of nasal mucosa for in vitro studies simulating nasal drug transport [27]. The cells were cultured in 75 cm^2^ flasks in complete minimum essential medium (MEM) containing 10% (*v*/*v*) fetal bovine serum (FBS), 1% *v*/*v* nonessential amino acid solution and 1% Penicillin/Streptomycin (PEN/STREPT) antibiotic. The culture was incubated at 37 °C with 95% air humidity and 5% CO_2_ atmosphere in a Nuaire (Caerphilly, UK) airflow incubator. The medium was changed 3 times a week until the cells reached confluence. Cells were regularly split at 80–90% confluency by a 5 min trypsinization (trypsin/EDTA) treatment.

Cytotoxicity assays were conducted using the human nasal septum carcinoma cell line RPMI 2650 and performing an MTT [3-(4,5-dimethylthiazol-2-yl)-2,5-diphenyltetrazolium bromide] colorimetric assay [28,29]. CUR and QU suspension, CQ_NE− and NE−, CQ_NE+, NE+, CQ_NEgel, and NEgel were all tested. To carry out the MTT cytotoxicity studies, RPMI 2650 cells in the 31–42 passage range were seeded in 48-well plates with an initial density of 1 × 10^7^ cell/mL and grown for 48 h in incubator to allow cell sedimentation and the complete cover of the well plate area. Concentrations from 0 to 164 μM and 0 to 131 μM were tested for QU and CUR, respectively, by direct dilution in cell culture medium of CQ_NE−, CQ_NE+ and CQ_NEgel. The cells were treated with the formulations for 4 h. At the end of incubation time, the supernatant was removed, and the cells were washed with 300 μL of PBS to remove any residue of NE formulations from the cell monolayer surface. Then cells were treated with MTT solution 1 mg/mL and incubated for further 2 h at 37 °C. Next, MTT was removed, and 200 μL/well of DMSO were added to dissolve the violet-colored metabolite. The plates were shaken in Orbital Shaker (SO3, Stuart Scientific, Cole-Parmer Ltd., Stone, UK) for 15 min at 150 rpm. The contents were pipetted and transferred to new plates and the absorbance was measured at 570 nm using a microplate reader (Spark 10 M, Tecan, Milan, Italy). Absorbance values were considered directly proportional to cell viability, and percentage cell viability was calculated by comparison to control values obtained for untreated cells.

### 2.5. Permeation Experiments across an In Vitro Model of Nasal Mucosa

For permeation experiments, human RPMI 2650 cells’ pseudo-monolayers were used as reported previously by Pozzoli and co-workers, cultivated in air–liquid interface conditions [30].

#### 2.5.1. Seeding on Transwell™ Inserts

The suspension of human RPMI 2650 epidermoid carcinoma cells was seeded on Transwell™ (12-well plates, area 1.12 cm^2^, 0.4 μm pore size) polyester inserts for the permeation experiment. Before the seeding, the Transwell™ inserts were coated with a 1.5 mg/mL collagen solution to increase the cells’ adherence to the membrane. Firstly, from the stock bovine collagen (5 mg/mL, Gibco™, Thermo Fisher Scientific, Waltham, MA, USA), a 3 mg/mL intermediate solution was prepared by dilution using glacial acetic acid 20 μM. This solution was diluted 50:50 *v*/*v* with absolute ethanol to prepare the final collagen solution and favor the rapid evaporation of the solvent. Transwell™ inserts were coated with 50 μL per well of collagen 1.5 mg/mL and kept in sterile conditions for 24 h until completely dry at 37 °C.

In order to establish the air–liquid interface (ALI) model, 1.5 mL of pre-warmed medium was added to the basolateral chamber, and 250 μL of cell suspension was seeded onto the collagen-coated Transwell™ polyester inserts at seeding concentration of 2.5 × 10^6^ cells/mL. The plate was incubated at 37 °C with 95% air humidity and 5% CO_2_ atmosphere for 24 h. After the 24 h of incubation, under submerged conditions, the apical volume of DMEM media was removed to allow cell cultivation under air–liquid interface (ALI) conditions for 14 days and to induce cell differentiation, leading to the formation of a cell layer expressing tight junctions and mucus suitable for permeation experiments. The culture medium from the basal chamber was removed and replaced with fresh medium every 2 days up to 14 days.

#### 2.5.2. Transepithelial Electrical Resistance (TEER) Measurements

To evaluate the integrity of the cell layer, the transepithelial electrical resistance measurement was used. TEER is a well-known procedure to quickly evaluate the integrity of epithelial cell layers. This is a non-invasive and harmless technique reflecting the ionic resistance across the cell layer during the various stages of growth and differentiation [31]. To evaluate the monolayer’s integrity and suitability for the transport studies, the TEER measurements of the RPMI 2650 cell layers were measured on the first and 14th day of culture, to ensure the formation of the cellular monolayer, using an EVOM1 epithelial Volt/Ohm meter (World Precision Instruments Inc., Sarasota, FL, USA). Since the TEER is affected by the pore size and density of the insert membranes, the measurement procedure includes measuring the blank resistance (R_BLANK_) of the membrane alone (without cells) and measuring the resistance across the cell layer on the insert membrane (R_TOTAL_). The cell layer specific resistance (R_CELLS_), in units of Ω, can be obtained as follows (Equation (8)):

(8) $$R_{\mathrm{CELLS}}\left(\Omega \right)=R_{\mathrm{TOTAL}}-R_{\mathrm{BLANK}}$$

TEER values are typically reported in units of Ω·cm^2^ and calculated as reported (Equation (9)):

(9) $${\mathrm{TEER}}_{\mathrm{REPORTED}}=R_{\mathrm{CELLS}}\left(\Omega \right)\times M_{\mathrm{AREA}}\;\left({\mathrm{cm}}^{2}\right)$$

#### 2.5.3. Curcumin and Quercetin Permeation across RPMI2650 Cells

The permeation experiments were carried out 14 days after seeding RPMI 2650 cells in Transwell™ inserts and growing them under ALI conditions. In particular, cells were treated with the three loaded CUR/QU_NEs prepared (CQ_NE−, CQ_NE+ and CQ_NEgel).

Before the treatment, the DMEM medium in the Transwell™ basal chamber (acceptor) was removed from each well and replaced with 1 mL of Hank’s Balanced Salt Solution (HBSS) at pH 5.5, to avoid proteins interference on the analytical method for drugs quantification. Amounts of 500 µL of the formulations (CQ_NE−, CQ_NE+, and CQ_NEgel) diluted 1:1 *v*/*v* in HBSS pH 5.5 (i.e, 250 µL of pure formulation) were added on the apical chamber (donor) of Transwell™ inserts. Plates were incubated at 37 °C with 95% air humidity and 5% CO_2_ atmosphere. Once an hour, for up to 4 h, 150 μL of liquid were taken from the acceptor compartment and 150 μL of fresh and pre-warmed HBSS was added to refill.

After 4 h of experiment, the formulations were collected from the apical compartment. Wells were rinsed with 500 μL of HBSS to remove any residue of formulation from the cells surface and added to the formulation collected from the well. Formulations collected from the apical chambers were treated as described in the Section 2.3.2 for NEs quantification, prior to HPLC analysis. Finally, the cells were detached from the membrane of each insert by adding 300 μL of acetonitrile and scratching carefully. All recovered samples (permeated cells and donor compartment) were analyzed by HPLC for CUR and QU content. Experiments were carried out in triplicate for each treatment condition.

## 3. Statistics

The statistical analyses were performed with Prism 7 software (GraphPad Software, San Diego, CA, USA). Each experiment was run at least in triplicate and results were expressed as mean values with the standard deviation, if not diversely specified. The results were analyzed using Student’s t-test (indicated as *t* in figure legend) for unpaired samples when comparing two groups or ordinary one-way ANOVA (indicated as An in figure captions) to compare more than two conditions, applying Tukey post hoc test for multiple comparisons.

## 4. Results

### 4.1. Preparation and Characterization of the Nanoemulsions

The nanoemulsions were prepared, enriching the oil phase with fish oil with the aim of taking advantage of both its solubilizing properties of CUR and QU and the neuroprotective properties of the polyunsaturated fatty acids contained in it. In fact, fish oil contains omega-3 fatty acids known to contribute to neuroplasticity and able to prevent the oxidative stress produced by reactive oxygen species, features considered beneficial in the prevention of neurodegenerative processes [32].

The mean particle size of the NEs obtained ranged from 102 nm to 131 nm, with the encapsulation of quercetin and curcumin. The particles’ sizes increased significantly due to encapsulation of the drugs from 102 to 119 (for NE−) not affecting the mean particle size (for NE+), while in the nanodroplets the size of the gel-modified NE increased to 244 nm when the natural compounds were encapsulated (Table 3).

A summary of the physicochemical properties, CUR and QU content, recovery, and entrapment efficiency of the positively charged, negatively charged, and gel-modified NEs are presented in Table 4. The drug content and entrapment efficiency (EE%) were determined with an HPLC with detection (LOD) and quantification (LOQ) limits 0.138 and 0.461 μg/mL for CUR and 0.074 and 0.248 μg/mL for QU, respectively. The maximal relative standard deviation values obtained were 2.57% for QU and 3.00% for CUR.

The CUR and QU content showed that it was possible to encapsulate approximately 0.60 mg/mL of CUR and 0.75 mg/mL of QU in drug loaded NEs. The entrapment efficiency values were higher than 99% for all the formulations (Table 4).

### 4.2. Nanoparticle Tracking Analysis

**Figure 3:**
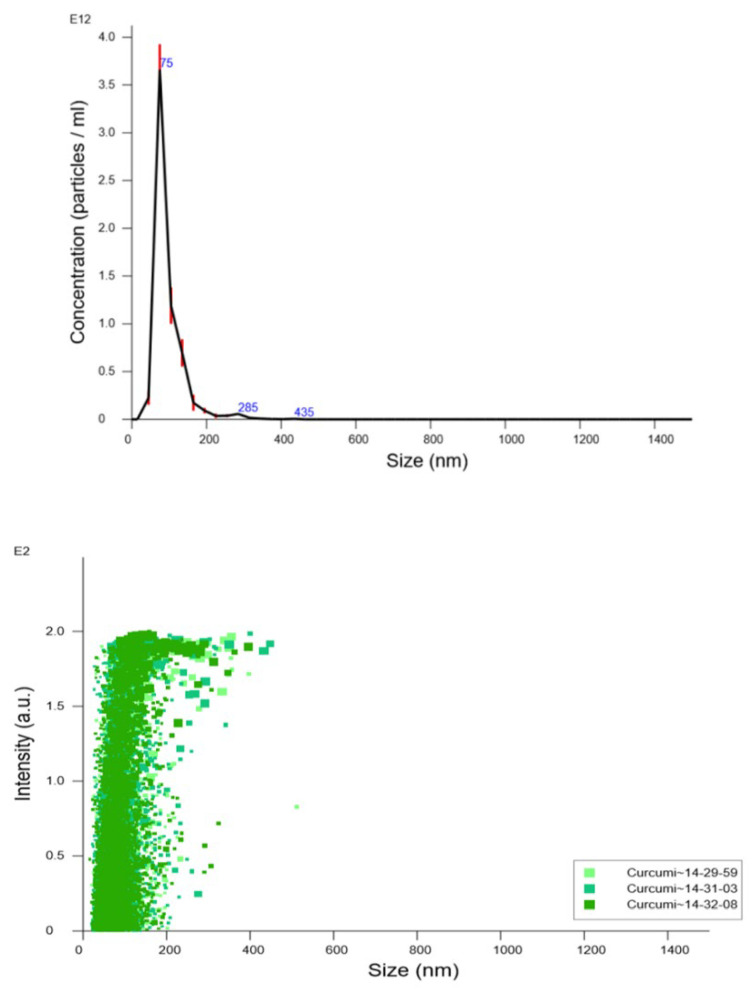
Particle size distribution vs. nanoparticle concentration by number (upper panel) and vs. intensity of scattered light obtained (lower panel) by nanoparticle tracking analysis. Particle size distribution is expressed as average and standard error of the mean of nanoparticle concentration.

NTA results obtained for blank negatively charged nanoemulsion (NE−) are shown in Figure 3. This nanoemulsion was selected as highly representative of all the other formulations since, in some way, all the others are derived from its basic composition. The particle size distribution by number showed that the main peak was present at 96.4 ± 1.8 nm and 90% of the particles were smaller than 142.7 ± 2.9 nm. Furthermore, the total particle concentration was calculated, the concentration being 6.21 ± 0.32×10^12^ particles/mL.

### 4.3. Nanoemulsions’ Stability

In a previous work, we already investigated the stability of quercetin and curcumin in a saline buffer at pH 7.4 and controlled temperature of 37 °C. A high instability of the natural compounds at the tested conditions was observed [20]. Here, we investigated the possible stability of the CUR- and QU-loaded NE formulation under refrigerated conditions for up to 120 days. In these conditions, the nanoemulsions’ size and surface charge did not evidence significant modifications over time (data not shown). Concerning CUR and QU content, when formulated in the herein proposed CQ_NE− formulations, the compounds remained stable for over 60 days when stored in refrigerated conditions (see Supplementary Materials Table S1). Interestingly, the stability of the natural compound was not optimal in the positively charged nanoemulsions (CQ_NE+), with QU and CUR contents already reduced after a couple of weeks of storage (see Supplementary Materials Table S2).

**Figure 4:**
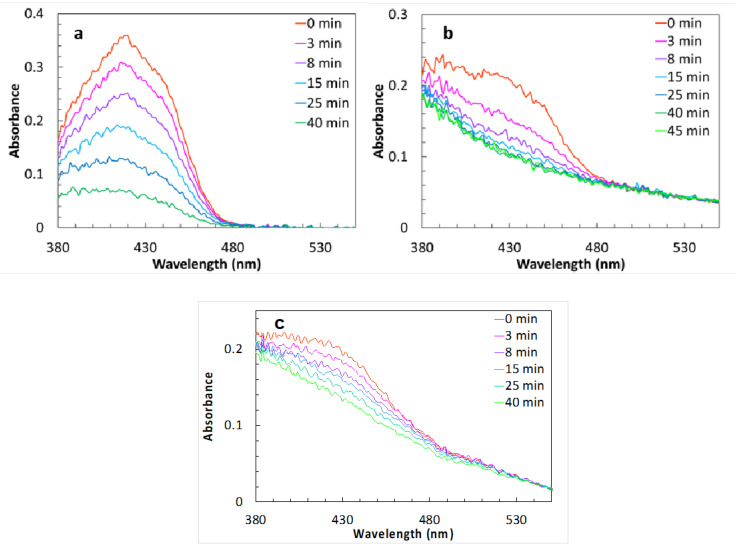
Absorption spectra of a curcumin 10^−4^ M solution of acetonitrile (**a**), of CQ_NE+ (**b**), and of CQ_NE− (**c**), diluted 1:1000, recorded at increasing exposure time.

Having evidenced a protection of the natural compounds in terms of stability in an aqueous medium, it was decided that we should assess if the nanoemulsion formulation could also impart a better photostability. Absorption spectra (Figure 4) are collected at different times of light exposure, both in acetonitrile solution and in suspension (spectra are noisy as the light intensity reaching the detector is very weak due to the small aperture of the cuvette and the low intensity of lamp below 380 nm). The absorption band of curcumin is clearly distinguishable in all the spectra, reaching a maximum at ≈420 nm. In the case of the solution, the decrease in the absorbance (A) at 420 nm during the time can be fitted with a curve of equation A = A_0_ + A_1_ · e^−k·t^, with k = 0.05 min^−1^ (A_0_ = 0.04, A_1_ = 0.31). This suggests a first order (monomolecular) process.

In the emulsions, the absorbance at 420 nm showed a single exponential decrease, as in the solution, but this was faster, with k = 0.22 min^−1^ in the positively charged samples and 0.1 min^−1^ in the negatively charged ones. This difference might be due to interactions between the curcumin and the surfactants at the surface of the micelles. The tail due to scattering was unaffected by irradiation, suggesting that only curcumin, dissolved in the micelles, is photobleached. These results suggest that the nanoemulsion does not protect the natural compounds from photodegradation, and that compounds should be stored in the dark.

### 4.4. Nanoemulsions’ Interfacial Properties Investigation by Electron Paramagnetic Resonance

All the systems were measured in the presence of 5-DSA or 16-DSA in order to obtain information about the dynamics of the nanoemulsion interfacial surface at two different depths of the surfactant monolayer.

#### 4.4.1. 16-DSA

The obtained results of the rotational correlation time (τ_R_) and parameter S (S) of 16-DSA in the empty and CUR- and QU-loaded systems are showed in Table 5. The EPR spectra of 16-DSA in the empty and loaded nanoemulsions are presented in the Supplementary Materials (Figure S1).

The rigidity of the membrane (parameter S) remained unaltered in all cases and the values indicate a system with a very flexible surfactant monolayer at that depth.

#### 4.4.2. 5-DSA

The obtained results of the rotational correlation time (τ_R_) and parameter S (S) of 5-DSA in the empty and loaded systems are showed in Table 6. The EPR spectra of 5-DSA in the empty and loaded systems are presented in the Supplementary Materials (Figure S2).

The comparison of the τ_R_ values of the two spin probes (Table 5 and Table 6) clearly indicates that the environment close to the polar heads of the surfactants (case of 5-DSA spin probe) makes the movement of the spin probe slower in all cases.

### 4.5. Nanoemulsions’ Antioxidant Effect

The antioxidant activity of empty and loaded high-energy NEs were analyzed using the EPR technique and the hydrophilic free radical Tempol (Table 7).

In the case of the loaded systems, the encapsulated compounds increased the antioxidant activity of the formulation from the first minute of the reaction. However, the standard deviation values obtained for the natural-compounds-loaded NE presented a higher value most likely related to the variability in the content of curcumin and quercetin in different batches. Nevertheless, the CUR- and QU-loaded positive nanoemulsions showed a higher free radical scavenging effect compared to the corresponding negative NEs.

### 4.6. Cytotoxicity on Human Nasal Cells

The three produced NEs were evaluated by MTT against the nasal cells line RPMI 2650, established as a suitable in vitro model of the nasal epithelium for the development of nasal products [30].

**Figure 5:**
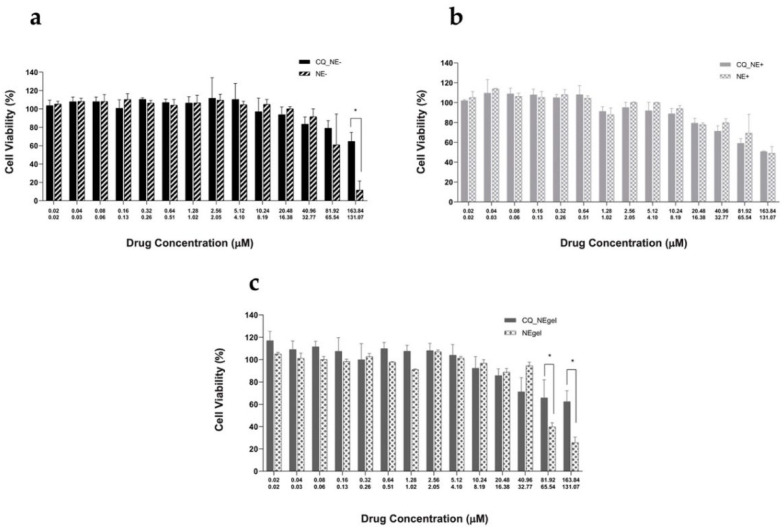
RPMI 2650 cells treated for 4 h with CQ_NE− (**a**)**,** CQ_NE+ (**b**), and CQ_NEgel (**c**), with increasing concentrations of the drugs and equivalent amounts of blank nanoemulsions. Graphs display cells’ viability expressed as a percentage in comparison to untreated cells. Drug concentrations for treatment with QU and CUR are 0–164 μM and 0–131 μM, respectively. * *p* < 0.05, *t.*

In Figure 5, the cell viabilities of RPMI 2650 following their treatment with different drug concentrations of CUR- and QU-loaded NEs (CQ_NE−, CQ_NE+, CQ_NEgel) are compared with the corresponding blank NEs (NE−, NE+, NEgel).

In Figure 5a, the cell viability percentages following treatment with increasing drug concentrations of QU (0–164 μM) and CUR (0–131 μM) loaded in negatively charged NEs (CQ_NE−) and the values obtained after treating cells with the corresponding blank formulation (NE−) are shown.

As illustrated in Figure 5b, in the case of positively charged NEs, there is no significant difference in cell viability between the drug-loaded and the corresponding blank NEs (*p* > 0.05, *t*).

In Figure 5c, the cell viability percentage following treatment with increasing drug concentrations of QU- and CUR-loaded and gelled negatively charged NEs (CQ_NEgel) and the corresponding values obtained using the corresponding blank formulation (NEgel) are shown.

### 4.7. Permeation Experiments on a Nasal Epithelium Cell Model

The ability of all the developed NEs to promote QU and CUR absorption through the nasal mucosa was investigated using a mucus-producing nasal epithelium model following a previously described experimental set-up employing the human nasal cell line RPMI2650 [33]. RPMI 2650 were grown in ALI for 14 days prior to the experiment to favor the formation of a cellular monolayer tissue. To verify the formation of the tight junction characteristic of the nasal epithelial tissue, the transepithelial resistance (TEER) was measured on day 1 (36 Ω·cm^2^) and day 14 (88 Ω·cm^2^).

**Figure 6:**
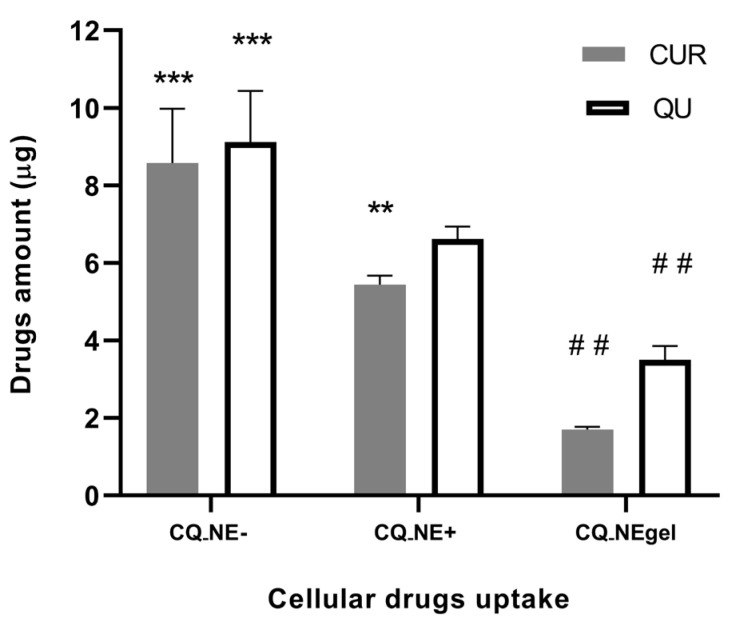
Mass (µg) of CUR and QU uptaken within RPMI 2650 epithelial cell line cultivated on Transwell^®^ inserts after treatment with CQ_NE−, CQ-NE+ and CQ_NEgel nanoemulsions. ** *p* < 0.01 compared to CQ_NE−, *** *p* < 0.01 compared to CQ_NE+, and ## *p* < 0.01 compared to CQ_NE+, An.

The graph shown in Figure 6 illustrates the results of NE transport studies across RPMI 2650 cells grown in air–liquid interface using Transwell^®^ inserts. In reality, in the basolateral chamber (acceptor compartment), it wasn’t possible to quantify either CUR or QU.

Contrarily, an uptake of the CUR and QU loaded in the NEs was evidenced according to the formulation properties in the cell monolayer. The mass of CUR taken up by the cells after 4 h incubation was about 8.5 μg for CQ_NE−, 5.4 μg for CQ_NE+ (significantly different compared to the negative NE, *p* < 0.01, An) and 1.8 μg for CQ_NEgel. QU followed the same trend, and QU uptake was 9 μg, 6.5 μg, and 3.5 μg for negative, positive, and gel formulations, respectively.

The percentages of total drug recovery for the whole experiment were: 72% of CQ_NE−, 67% of CQ_NE+, and 78% of CQ_NEgel for CUR; and 65% of CQ_NE−, 64% of CQ_NE+, and 58% of CQ_NEgel for QU.

## 5. Discussion

In the present study, the NEs containing CUR and QU were prepared by high-pressure homogenization. To prepare the formulations with different surface charges, a study comparing the incorporation of cetalkonium chloride (CC) or benzalkonium chloride (BC) into the original negatively charged formulation was performed (see Supplementary Materials Table S3). The positive formulation optimization was carried out by varying the amount of CC and BC, and the CC was the cationic agent selected for the production of the cationic NE in the concentration of 0.0175% (NE-P) since it has been demonstrated to be able to invert the charge of NE at lower concentrations compared to BC. Moreover, CC is a well-tolerated and widely used cationic surfactant [34].

The developed formulations showed, in all cases, nanoemulsions with a mean particle size below 150 nm, demonstrating no significant impact of curcumin and quercetin encapsulation. The only exception was the case of the entrapment of the two natural compounds in the gel-modified negative NE, where the particle size was almost doubled, probably due to the influence of both the gelling agent and the entrapped compounds that could have promoted the aggregation/coalescence of nanodroplets. The nanoemulsions displayed monodisperse particle size distributions, as indicated by the relatively low values of the polydispersity index (PDI < 0.3) [35].

The slightly lower drug recovery evidenced for CUR compared to QU could possibly be due to drug loss in the preparation process, either as a consequence of chemical degradation or adsorption to the glassware. However, when formulated in the nanoemulsions (CQ_NE− used as platform), the CUR and QU content remained above 95% for at least 60 days under refrigerated conditions. On the other hand, it appeared that photostability could still be an issue for the nanoemulsion formulations and could be at least partially responsible for the reduced drug recovery observed.

In order to confirm the particle-sizing data obtained by DLS, the particle size distribution, and particle concentration of the NE used as “platform” for the others were measured using NTA. NTA is a relatively new investigation technique that offers direct and real-time visualization, sizing, and counting of nanoparticles, allowing high-resolution particle size distributions to be obtained [28,36]. A smaller NTA average particle size in comparison with DLS results was expected due to the differences between the methods. In fact, while NTA gives a number distribution, DLS provides an intensity weighed average, and the scattered light intensity is much higher for large particles. Hence, NTA provides complementary information to both DLS and electron microscopy. In fact, as it follows individual particles, it enhances the resolution of polydisperse particle populations which are usually not so well resolved by DLS. The technique still operates on a statistically significant number of particles, larger than for microscopy, although not determining their morphology [37]. The particle size distribution in NTA confirmed a narrow size distribution for the NE formulation proposed.

The surfactants’ interfacial dynamics were investigated in the presence of 5-DSA and 16-DSA spin probes by electron paramagnetic spin resonance. The spectra obtained using 16-DSA indicated that, upon the addition of the cationic surfactant and of the CUR and QU, no relevant alterations occurred in the surfactant membrane at the interface of the nanoemulsion. The comparison of the τ_R_ values of the two spin probes showed that the environment close to the polar heads of the surfactants makes the movement of the spin probe 5-DSA slower in comparison to the 16-DSA spin probe, as indicated by the lower τ_R_ values. These results evidenced for all nanoemulsions could be linked to the surfactant packing in the monolayer of the NEs. The inner part of the surfactant membrane is less compact in contrast to the outer one, close to the polar heads, which creates a hindrance to the movement of the amphiphilic spin probe. The addition of the cationic surfactant in the NEs induces slight alterations in the region close to the polar heads of the surfactants as indicated by the lowering τ_R_ values. The cationic surfactant localized close to the polar heads of lecithin and Solutol^®^ creates a disturbance in the surfactant layer allowing for a quicker movement of the spin probe 5-DSA. The 16-DSA-spin-probe-related values on the contrary were not affected, meaning that the cationic surfactant did not affect the inner packing of the surfactant membrane. It has been reported previously that BC decreases the macro-viscosity of nanoemulsion systems owing to ionic aggregation; in fact, that could explain the increased mobility of the 5-DSA spin probe in the case of the positive NEs [38].

Also, after the incorporation of CUR and QU in the NEs, the movement of the 5-DSA spin probe was affected. CUR and QU are highly lipophilic bioactive molecules. The molecules’ entrapment appears to affect the spin probe’s movement following a specific pattern. The entrapment of CUR and QU increased the τ_R_ values for both negative and positive nanoemulsions, indicating an effect on the surfactant interface. As those alterations were not observed in the case of the spin probe 16-DSA, the results suggest that the entrapped compounds are localized in between the hydrophobic surfactant chains and the hydrophilic polar heads, but closer to the latter. In a previous study, CUR was also located in the surfactant membrane of a low-energy NE obtained using lecithin and a high-HLB-value surfactant similar to the present case [39]. However, since in the present study we have not studied the localization of each compound separately, we can assume that both CUR and QU are located at the interface, even if, based on the logP values of the two substances, QU is the less hydrophobic of the two compounds (CUR: 4.12 and QU: 2.16). The presence of Solutol^®^ in the membrane creates an environment where both antioxidants have higher solubility in comparison to the dispersed oil phase. As QU is “friendlier” to water, it can be assumed that its position is closer to the polar heads of the surfactants than the corresponding position of CUR.

The addition of CUR/QU in the positive system increased the τ_R_ value of the 5-DSA more in comparison to the corresponding value of the negative system. This was attributed to the addition of the cationic surfactant to the system. The addition of this surfactant in the formulation could further increase the solubility of the lipophilic antioxidants. The antioxidants tend to be localized at the region where they can be solubilized better. This location is where the surfactants coexist, meaning close to the polar heads of the surfactant molecules. The 5-DSA can “sense” a higher concentration of CUR/QU in its environment in the case of positive nanoemulsions.

The blank nanoemulsions, both negative and positive, showed relatively low antioxidant activity. As Pan and collaborators (2013) suggested that lecithin has antioxidant activity, it is possible that lecithin is the component that provide the antioxidant activity shown by the empty nanoemulsions towards the free radical Tempol, possibly in combination with the dispersed oil phase [40].

In the case of the CUR- and QU-loaded nanoemulsions, the addition of the natural compounds increased the antioxidant activity of the formulation from the first minute of the reaction. However, antioxidant activity appeared to be slightly variable due to the interbatch differences in CUR and QU content. The NE manufacturing method, including heating (70 °C) and high-pressure processing (1000 bars), probably altered the initial antioxidant properties of the entrapped compounds on some level, explaining the obtained difference.

Biocompatibility is a prerequisite for pharmaceutical formulations. In view of nose-to-brain delivery, the nasal epithelial layer represents the body’s first barrier, and materials harming the mucosal epithelium constitute a potential health risk [41,42,43]. MTT represents one of the most common tests to evaluate cellular toxicity. Since the cellular reduction of the MTT reagent can be catalyzed only by living cells, it is possible to quantitatively determine the percentage of living cells 44]. The cytotoxicity studies on the human nasal cell line RPMI2650 of the negative NEs loaded with CUR and QU showed that, at the highest concentrations, the blank formulation reduced the cellular viability to just 10%, while cells treated with the CUR- and QU-loaded formulation showed a significantly higher cellular viability of near 70% (*p* < 0.05, t). This is an interesting result, since it has been reported that NEs, and especially their surfactant components, can have a negative impact on cell viability [45]. However, it seems that in the case of the negative nanoemulsion the two natural compounds studied here exert a protective action on cells, maintaining cell viability at high levels at all the concentrations tested. In the case of positively charged NEs, for both the blank and the CUR- and QU-loaded NEs, there occurred a concentration-dependent decrease in nasal cell viability, with around 50% cells viable at the highest concentration tested. The results suggest that CUR and QU loading in the case of positive NE does not protect cells against cytotoxic effects. This could be explained by the higher toxicity generally reported for positively charged surfactants on cells and tissues [46]. Indeed, in the case of nasal delivery, cationic surfactants used as preservatives in liquid formulations have been evidenced, in vitro, to damage the cell membranes of human nasal epithelial cells and to promote the permeation of desmopressin across animal nasal tissue ex vivo [47,48]. In the case of the NEs formulated with the in situ gel agent, the drug-loaded formulation (CQ_NEgel) showed again a protection of cell viability when compared to the blank NE. In fact, at the higher concentration tested, the blank NE led to a reduction in the cellular viability in about 25% of the untreated cells, while the drug-loaded gel NE led to only a 40% reduction in cell viability (60% of cells viable) at the higher concentration. The slightly lower protection by CUR and QU observed in comparison to the non-gel formulation probably has to be ascribed to the viscosity formulation.

It is worth mentioning that the results obtained with the MTT test could lead to an underestimation of cellular viability, since the test is based on cells’ metabolic activity. Furthermore, the reduction in cellular viability at the highest concentration could not only be due to the NE components but also to the physical properties of the formulation and in particular to low dilution. In fact, at a high NE concentration, direct mechanical damage or cell suffocation could have occurred.

The human epidermoid carcinoma epithelial cell line RPMI 2650, when grown in air–liquid interface (ALI) conditions, develops tight junctions and produces a mucus layer on the apical surface. These culture conditions allow us to more closely simulate the in vivo conditions found in the nasal mucosa and this in vitro model of the nasal mucosa is suitable for studies of deposition and the permeation of nasally administered formulations [49,50]. The TEER indicated the formation of a mature epithelial tissue on the 14th day of culture (88 Ω·cm^2^).

In permeation studies, no CUR or QU was detected in the basolateral chamber (acceptor compartment) as the permeated drug concentrations were lower than the LOD and LOQ values of the developed method. This could be explained in two ways: firstly, considering the extremely low solubility of the two compounds in water, as plain CUR is practically insoluble in water [51,52], and QU also has very poor water solubility [53]; secondly, taking into account their instability in aqueous media. In fact, significant reductions in the concentrations of both drugs were observed in the phosphate buffer at pH 7.4 at a temperature of 37 °C already after 2 h (QU 28.0 + 2.1% and CUR 79.5 + 2.1% of the initial concentration) [20]. Hence, CUR’s and QU’s properties make the possibility of dosing these natural drugs in the acceptor compartment after permeation very unlikely.

It was, however, possible to determine the intracellular concentration of the drugs, probably related to the nanoemulsions’ uptake by the nasal cells. The cellular uptake of CUR and QU was reduced for CQ_NEgel compared to the positively and negatively charged NEs (*p* < 0.01, A), probably because of the increased viscosity of the matrix surrounding the colloidal dispersion. In fact, droplets and drugs must diffuse through the liquid vehicle before they can be in contact with cells, and a viscous formulation could delay the rate and extent of cell uptake. Despite positively charged nano-formulations having been reported to enhance drug permeation across several epithelial barriers [34,54], in the present work, NE+ formulation showed a reduced cellular internalization of CUR and QU compared to the negatively charged formulation. This could be explained, considering that the mucus layer is well-known to affect particle diffusion and drug permeation across mucosal tissues [55,56]. In particular, the polyanionic mucin glycoproteins are the main proteins responsible for interaction filtering, so, unsurprisingly, positively charged nanoparticles are typically trapped in the mucus network [57,58]. In the applied nasal mucosa model, RPMI 2650 cells cultivated for 14 days in an air–liquid interface produced a continuous mucus layer covering their apical surface, providing a barrier comparable to previously reported nasal epithelium models and, more importantly, to excised human nasal mucosa [30]. It appears likely that cationic nanoemulsions in the presence of the mucus layer were prevented from interacting with the underlying cells, while the negatively charged nanoemulsions were able to cross the barrier more efficiently.

The relatively low recovery of the two drugs in these experiments is again likely due to the drug instability in aqueous environments, or even in the cellular environment, once it is not protected by the nanoemulsion. The results suggest that, indeed, drug degradation could have occurred during the permeation experiments. It is reported that CUR and QU are highly susceptible to several types of degradation, such as photodegradation and pH-dependent degradation [59,60]. Since the final mass balance of both compounds do not correspond to the theoretical amount of formulation initially added, it is highly likely that a degradation of the free drugs is occurring after permeation.

## 6. Conclusions

The production of nanoemulsions loaded with CUR and QU proved possible, and the method used was adapted to obtain nanocarriers of appropriate size for nasal administration, as well as a high concentration of CUR and QU, such as to allow a possible anti-inflammatory effect for the prevention of neurodegenerative diseases. Three different NEs, one negatively charged, one positively charged, and one with increased viscosity (CQ_NE−, CQ_NE+, and CQ_NEgel), were tested.

NEs were toxic for RPMI 2650 cells only at the highest concentrations tested, and the formulations loaded with the drugs also showed a protection of cellular viability compared to the blank formulations, with the exception of the positively charged NEs because of the higher toxicity generally reported for positively charged tensioactives on cells.

RPMI 2650 cells used in this work were confirmed to be an appropriate alternative to excised nasal mucosa for the in vitro evaluation of nasal drug permeation. The multilayer cell model showed similar characteristics to excised mucosa, including TEER values. A significant cellular uptake was evidenced in the permeation experiment, despite no drug permeation being detectable, probably as a consequence of the low solubility of the two compounds in water and the instability of the drugs in aqueous solution. CUR and QU cell uptake was lower for CQ_NEgel, probably because of the formulation’s viscosity, while uptake was highest for the negatively charged NEs.

Despite some limitations, such as the absence of ciliated cells that simulate the mucociliary clearance present in vivo, this work demonstrated that RPMI 2650 cells in an air–liquid interface culture can be used as a first screening tool in the preclinical evaluation and comparison of nanocarriers for the IN delivery of drugs, and that nanoemulsion formulations and physicochemical properties can affect the cytotoxicity and cell uptake of natural compounds such as curcumin and quercetin.

## Figures and Tables

**Table 1:** Composition of the nanoemulsions prepared.

Formulation	PEG 660 Stearate (% *w*/*v*)	Castor Oil (mg)	Fish Oil (mg)	Egg Lecithin (mg)	CUR (mg)	QU (mg)	Cetalkonium Chloride (% *w*/*v*)	Deacetylated Gellan Gum (% *w*/*v*)
NE−	1.5	2400	2400	1200	-	-	-	-
CQ_NE−	1.5	2400	2400	1200	45	45	-	-
NE+	1.5	2400	2400	1200	-	-	0.0175	-
CQ_NE+	1.5	2400	2400	1200	45	45	0.0175	-
NEgel	1.5	2400	2400	1200	-	-	-	0.5
CQ_NEgel	1.5	2400	2400	1200	45	45	-	0.5

**Table 2:** Structural and molecular formula of 5-DSA and 16-DSA.

Name (Acronym)	Structural Formula	Molecular Formula
5-doxyl stearic acid (5-DSA)	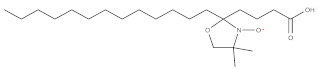	C_22_H_42_NO_4_
16-doxyl stearic acid (16-DSA)	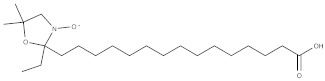	C_22_H_42_NO_4_

**Table 3:** Size, polydispersity index, and zeta potential of the nanoemulsions prepared.

Nanoemulsion	Size (nm)	PDI ^1^	ζ Potential (mV)
NE−	102.86 ± 1.80	0.183 ± 0.02	−25.7 ± 0.20
NE+	131.86 ± 0.80	0.246 ± 0.01	+6.4 ± 0.20
NEgel	134.87 ± 0.40	0.240 ± 0.03	−25.7 ± 0.2
CQ_NE−	119.43 ± 0.83	0.202 ± 0.02	−22.3 ± 0.15
CQ_NE+	131.00 ± 0.25	0.210 ± 0.01	+7.9 ± 0.24
CQ_NEgel	244.80 ± 2.40	0.191 ± 0.01	−29.1 ± 0.40

^1^ PDI, polydispersity index.

**Table 4:** Drug content, recovery, and entrapment efficiency.

Nanoemulsion	Curcumin Content (mg/mL)	Recovery Curcumin (%)	Quercetin Content (mg/mL)	Recovery Quercetin (%)	Entrapment Efficiency (%)
CQ_NE−	0.61 ± 0.01	81.33 ± 1.30	0.72 ± 0.01	96.00 ± 1.30	>99
CQ_NE+	0.62 ± 0.02	82.66 ± 2.60	0.71 ± 0.03	94.66 ± 4.00	>99
CQ_NEgel	0.61 ± 0.01	81.33 ± 1.30	0.72 ± 0.01	96.00 ± 1.30	>99

**Table 5:** Rotational correlation time (τ_R_), order parameter (S), and polarity of 16-DSA in the empty and loaded systems.

16-DSA	τ_R_ (ns)	S	α´_0_
NE−	1.46 ± 0.07	0.08 ± 0.01	14.27 ± 0.05
CQ_NE−	1.51 ± 0.07	0.08 ± 0.01	14.20 ± 0.03
NE+	1.40 ± 0.05	0.08 ≤ 0.01	14.36 ± 0.06
CQ_NE+	1.46 ± 0.06	0.08 ± 0.01	14.32 ± 0.03

**Table 6:** Rotational correlation time (τ_R_) and parameter S (S) of 5-DSA in the empty and loaded systems.

5-DSA	τ_R_ (ns)	S
NE−	6.74 ± 0.08	0.49 ± 0.02
CQ_NE−	6.97 ± 0.07	0.50 ± 0.01
NE+	6.37 ± 0.04	0.49 ± 0.01
CQ_NE+	6.70 ± 0.04	0.50 ± 0.01

**Table 7:** Scavenging effect of Tempol free radical (expressed as %) over time of nanoemulsions.

Time (Min)	NE−	CQ_NE−	NE+	CQ_NE+
2	8.3 ± 0.4	15.6 ± 5.2	5.3 ± 1.6	22.7 ± 6.7
5	8.9 ± 1.1	18.3 ± 5.3	6.8 ± 1.5	24.3 ± 5.8
10	9.8 ± 0.7	19.9 ± 4.5	8.1 ± 1.1	24.8 ± 5.2
15	10.4 ± 0.7	19.1 ± 5.9	8.1 ± 1.1	24.8 ± 4.9
20	10.8 ± 1.0	19.3 ± 5.8	7.7 ± 1.4	25.0 ± 4.8
25	10.5 ± 1.0	19.8 ± 5.3	8.5 ± 0.8	24.9 ± 4.9
30	10.5 ± 0.9	19.7 ± 5.2	8.9 ± 0.8	25.1 ± 4.7

## Data Availability

Data are available upon request to the corresponding author.

## Supplementary Materials

The following supporting information can be downloaded at: https://www.mdpi.com/article/10.3390/pharmaceutics14010194/s1, Figure S1: EPR spectra of 16-DSA in the empty and loaded systems. Experimental conditions were center field: 0.349 T, scan range: 0.01 T, gain: 2.24 × 103, time constant: 5.12 ms, modulation amplitude: 0.4 mT and frequency: 9.78 GHz. The measurements were conducted at room temperature. monolayer; Figure S2: EPR spectra of 5-DSA in the empty and loaded systems. Experimental spectra are plotted with solid lines and the corresponding simulated with red dotted line. Experimental conditions were center field: 0.349 T, scan range: 0.01 T, gain: 5.64 × 103, time constant: 5.12 ms, modulation amplitude: 0.4 mT and frequency: 9.78 GHz. The measurements were conducted at room temperature; Table S1: Size, polydispersity index and zeta potential of the formulations prepared with cetalkonium chloride and benzalkonium chloride; Table S2: Stability of Curcumin and Quercetin loaded nanoemulsion (CQ_NE−) stored at 5–8 °C; Table S3: Stability of Curcumin and Quercetin loaded nanoemulsion (CQ_NE+) stored at 5–8 °C.
