# Supplementary material for: In Vitro Evaluation of Curcumin- and Quercetin-Loaded Nanoemulsions for Intranasal Administration: Effect of Surface Charge and Viscosity
Source: Pharmaceutics. 2022 Jan 14;14(1):194. doi: 10.3390/pharmaceutics14010194 (PMC8779979; doi:10.3390/pharmaceutics14010194)
Supplement: Supplementary file 1 [file pharmaceutics-14-00194-s001.zip › pharmaceutics-1513813-supplementary.pdf]

pharmaceutics

Supplementary Materials: In Vitro Evaluation of Curcumin- and Quercetin-Loaded Nanoemulsions for Intranasal Administration: Effect of Surface Charge and Viscosity

Gustavo Vaz, Adryana Clementino, Evgenia Mitsou, Elena Ferrari, Francesca Buttini, Cristina Sissa, Aristotelis Xenakis, Fabio Sonvico and Cristiana Lima Dora

On Figure S1, the EPR spectra of 16-DSA in the empty and loaded systems are presented.

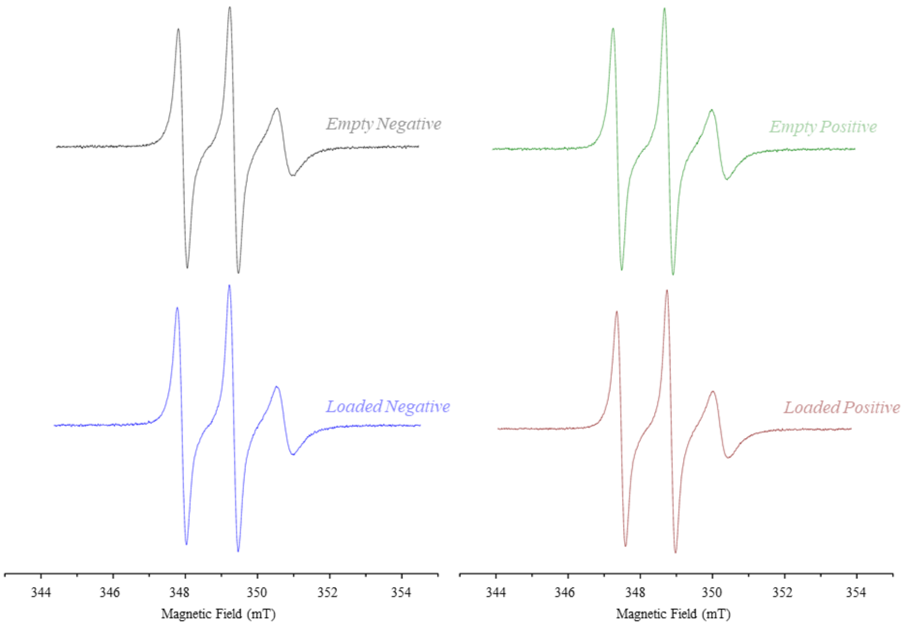

Figure S1. EPR spectra of 16-DSA in the empty and loaded systems. Experimental conditions were center field: 0.349 T, scan range: 0.01 T, gain: 2.24 × 103, time constant: 5.12 ms, modulation amplitude: 0.4 mT and frequency: 9.78 GHz. The measurements were conducted at room temperature. monolayer.

On Figure S2, the EPR spectra of 5-DSA in the empty and loaded systems are presented.

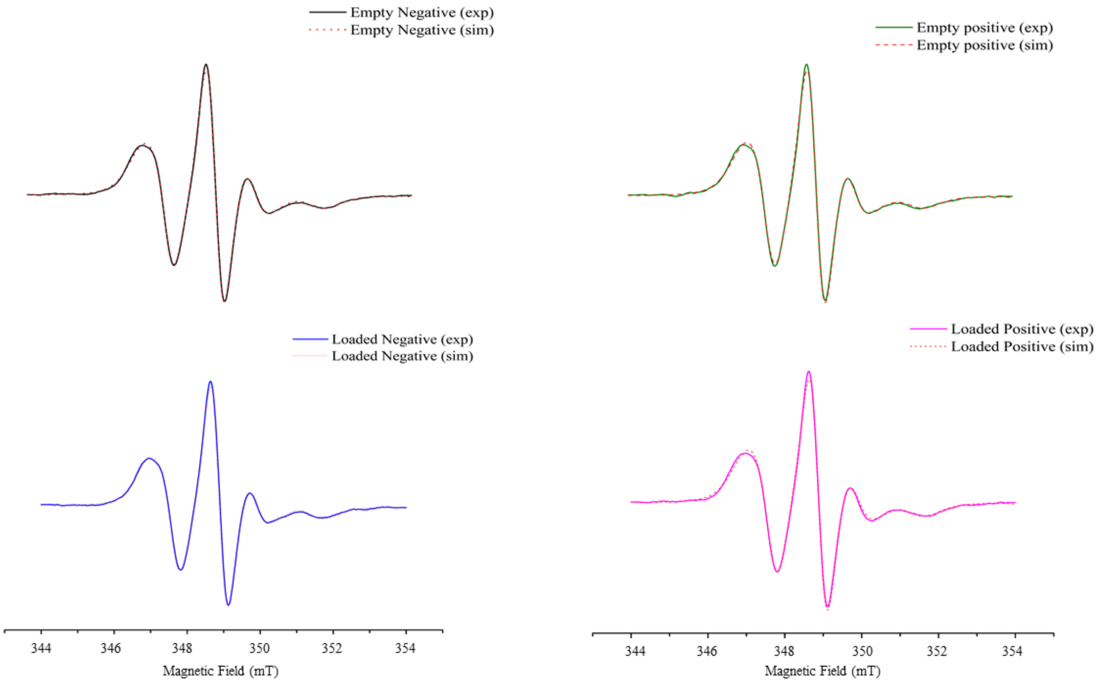

Figure S2. EPR spectra of 5-DSA in the empty and loaded systems. Experimental spectra are plotted with solid lines and the corresponding simulated with red dotted line. Experimental conditions were center field: 0.349T, scan range: 0.01 T, gain: 5.64 × 103, time constant: 5.12 ms, modulation amplitude: 0.4 mT and frequency: 9.78 GHz. The measurements were conducted at room temperature.

Concerning photostability of quercetin and curcumin when loaded in negatively charged nanoemulsions under refrigerated conditions results are presented in Table S2.

Table S1. Stability of Curcumin and Quercetin loaded nanoemulsion (CQ_NE−) stored at 5 °C–8 °C.

Days	Quercetin Average Content (%)	Quercetin SD	Curcumin Average Content (%)	Curcumin SD
0	100.0	-	100.0	-
30	99.9	2.8	99.9	2.5
60	95.6	2.5	98.6	2.9
120	36.7	0.8	45.1	1.7

SD: Standard Deviation, *n* = 3.

Table S2. Stability of Curcumin and Quercetin loaded nanoemulsion (CQ_NE+) stored at 5 °C–8 °C.

Days	Quercetin Average Content (%)	Quercetin SD	Curcumin Average Content (%)	Curcumin SD
0	100.0	-	100.0	-
7	96.9	2.7	83.3	3.0
14	97.0	2.6	85.9	2.4
30	69.4	4.1	72.7	5.9

SD: Standard Deviation, *n* = 3.

In the present work, a preliminary study comparing the incorporation of CC or BC into the original negatively charged formulation by varying the amount of CC and BC was performed (Table S3), and the effect was evaluated on the size, polydispersity index (PDI), and zeta potential.

Table S3. Size, polydispersity index and zeta potential of the formulations prepared with cetalkonium chloride and benzalkonium chloride.

**Nanoemulsion**	**Benzalkonium chloride (%)**	**Size (nm)**	**PDI [1]**	**ζ-potential (mV)**
Blank 1 – BC	0.0100	126.9 ± 1.02	0.208	−29.70 ± 0.21
Blank 2 – BC	0.0175	107.4 ± 0.46	0.189	−2.89 ± 0.18
Blank 3 – BC	0.0250	117.2 ± 1.12	0.207	+3.81 ± 0.28
Blank 4 – BC	0.0500	133.3 ± 0.43	0.217	+17.40 ± 0.15
Blank 5 – BC	0.1000	125.1 ± 1.11	0.190	+41.06 ± 0.41
	**Cetalkonium chloride (%)**			
Blank 1 – CC	0.0100	160.3 ± 1.34	0.250	−3.48 ± 0.32
Blank 2 – CC	0.0175	131.8 ± 0.85	0.246	+6.45 ± 0.21

[1] PDI, polydispersity index.
